# Longitudinal Survey of Fecal Microbiota in Healthy Dogs Administered a Commercial Probiotic

**DOI:** 10.3389/fvets.2021.664318

**Published:** 2021-06-21

**Authors:** Susan Ciaravolo, Lina María Martínez-López, Richard J. N. Allcock, Andrew P. Woodward, Caroline Mansfield

**Affiliations:** ^1^Department of Veterinary Clinical Sciences, Melbourne Veterinary School, The University of Melbourne, Werribee, VIC, Australia; ^2^Peninsula Vet, Emergency and Referral Hospital, Mornington, VIC, Australia; ^3^School of Biomedical Sciences, The University of Western Australia, Perth, WA, Australia; ^4^Faculty of Veterinary and Agricultural Sciences, The University of Melbourne, Werribee, VIC, Australia

**Keywords:** microbiota, probiotic, *Lactobacillus*, *Enterococcus*, *Bifidobacterium*, *Streptococcus*, synbiotic

## Abstract

The aim of this longitudinal microbiome study was to investigate the effects of a commercially available veterinary synbiotic product (Blackmore's® Paw DigestiCare 60™) on the fecal microbiome of healthy dogs using 16S rRNA gene microbial profiling. Fifteen healthy, privately-owned dogs participated in a 2-week trial administration of the product. Fecal samples were collected at different time points, including baseline (prior to treatment), during administration and after discontinuation of product. Large intra- and inter-individual variation was observed throughout the study, but microbiome composition at higher phylogenetic levels, alpha and beta diversity were not significantly altered after 2 weeks of probiotic administration, suggesting an absence of probiotic impact on microbial diversity. Administration of the synbiotic preparation did, however, result in transient increases in probiotic species from *Enterococacceae* and *Streptococacceae* families as well as an increase in *Fusobacteria;* with the fecal microbiota partially reverting to its baseline state 3-weeks after cessation of probiotic administration.

## Introduction

The contribution of the gastrointestinal (GI) microbiome to both GI and systemic health is a burgeoning field of research in both human and veterinary medical sciences. This complex bacterial (fungal, viral, and protozoal) community performs vital intestinal functions and a variety of other physiological roles (1). Not only does the GI microbiome contribute to normal function but may also contribute to disease states of the heart, kidneys, liver, nervous system and of course the gastrointestinal tract (GIT) (1–5). Because of the implications for systemic health, there is great interest in establishing what constitutes a “normal” or “healthy” GI microbiome, and what alterations or deficiencies result in susceptibility to, or occurrence of, a multitude of diseases (1, 4, 6).

The natural progression from this is to investigate pharmaceuticals and nutraceuticals that alter the GI microbiome, to be used in the prevention or treatment of disease or disease progression. Probiotics and prebiotics are currently utilized to restore and maintain a favorable balance of the GI ecosystem. The International Scientific Association for Probiotics and Prebiotics (ISAPP) define probiotics as “live microorganisms that, when administered in adequate amounts, confer a health benefit on the host”; this can include exogenous and indigenous microorganisms (7). Prebiotics are defined as “a selectively fermented ingredient that results in specific changes in the composition and/or activity of the GI microbiota, thus conferring benefits upon host health,” while synbiotic refers to preparations combining both pro- and pre-biotics “that beneficially affect the host by improving the survival and implantation of live microbial dietary supplements in the GIT” (8, 9). All are increasingly being used in veterinary practice, gaining popularity among pet owners and veterinarians alike, as over-the-counter food supplements (10).

Widespread mechanisms of probiotic actions include competitive exclusion of bacteria with pathogenic potential; production of essential micro and macronutrients [including short chain fatty acids (SCFA)]; promotion of junction stability between intestinal epithelial cells; normalization of perturbed GI microbiota; production of antimicrobial substances; and improvement of the digestion process (7, 11). Some mechanisms are widespread across taxonomic groups, while others are strain-specific (7). The effect of any probiotic depends on its metabolic properties, cell surface molecules, and secreted components (12). Certain biological effects of a probiotic may only be feasible if exerted by viable organisms that successfully survive the GIT and adhere to the intestinal epithelium, while others may be exerted by metabolites or components of bacteria that do not survive intestinal transit (7, 12, 13). The biological effects of probiotics may also vary depending on which host species the strains are isolated from (14, 15). Likewise, the recipient host intestinal microenvironment and its microbiota will also influence the effect. Knowledge about the effects and interactions of probiotic strains with the host gut microbiota are lacking and requires further investigation.

Systematic reviews in human medicine have not found clear evidence for an effect of probiotics on the gut microbiota in healthy individuals, with only 4/19 (21%) trials reviewed documenting alteration of the GI microbiota in healthy individuals administered probiotics (16, 17). Most studies, related to the use of probiotics in dogs, have assessed their utility in treating both acute and chronic enteropathies, but little is reported on their use in healthy animals as supplements (1, 18–23). Moreover, not all commercially available products have been evaluated scientifically to determine their effect, if any, in either healthy or diseased dogs. Blackmore's® Paw DigestiCare 60^TM^ has not previously been evaluated but contains bacterial strains which have reported probiotic effects or characteristics *in vitro* or *in vivo*. These include: *Lactobacillus acidophilus, L. rhamnosus, L. delbrueckii* subspecies *bulgaricus, L. plantarum and Enterococcus faecium* (7, 9). The prebiotics found in this preparation have also been previously reported to increase fecal short-chain fatty acids and enrich fiber-fermenting Firmicutes bacterial groups – although there is insufficient evidence to support any beneficial effect on microbiome function (24, 25).

In this study a commercially available multi-strain synbiotic veterinary product (Blackmore's® Paw DigestiCare 60^TM^), which has not previously been studied, was administered to healthy dogs with a goal to document any effects to the diversity and composition of the fecal microbiome at short term. Using high-throughput ion-torrent pyrosequencing the fecal microbiome was studied for changes, from baseline, during and after probiotic administration. We hypothesize that synbiotic administration would alter the fecal microbiome of healthy adult dogs for the duration of treatment, but changes would be transient and revert upon discontinuation of the supplement.

## Methods

### Study Dogs

Healthy dogs were recruited on a volunteer basis for participation in a prospective trial, through advertisement to University veterinary staff and veterinary students. Dogs were included in the study after a thorough physical examination and history found they were free of apparent clinical disease and up to date with routine vaccinations and ecto-/endo- parasite control. Criteria of exclusion included: dogs <1 year of age, detection of abnormalities on physical examination, history of signs of chronic disease, a recent history of corticosteroid, non-steroidal anti-inflammatory, proton pump inhibitor or antibiotic use within the last 3 months, or evidence of recent or chronic gastrointestinal disease (e.g., vomiting or diarrhea). Dogs were followed for 1 year following the study to ensure no disease developed.

This study was approved by and in accordance with the Animal Ethics committee of University of Melbourne (Animal Ethics Committee approval AEC #1413272.2), using National Health and Medical Research Council (NHMRC) guidelines. Owners gave written consent and were able to withdraw their animal from the study at any time.

### Probiotic Treatment

The product used, Blackmore's® Paw DigestiCare 60^TM^ (Australia), is labeled to contain per 2 grams of powder: 60 million colony forming units (CFU) as: *Lactobacillus acidophilus; L. delbrueckii* subspecies *bulgaricus; L. plantarum; L. rhamnosus; Bifidobacterium bifidum; Enterococcus faecium; Streptococcus salivarius* subspecies *thermophilus* combined with alfalfa grass, quinoa, spirulina and other legumes and cereals.

The probiotic was administered in its powdered form mixed with the animal's meal once per day for 14 consecutive days. Dose of the probiotic was based on the dog's weight, in accordance with the manufacturer's guidelines: 2 grams for dogs between 5 and 10 kg, 4 grams for dogs between 11 and 25 kg and 6 grams for dogs > 25 kg. All subjects were maintained on their typical commercial diet during the study period, water was supplied *ad libitum*. No dog was fed a raw meat diet. Owners were asked to record clinical signs of gastrointestinal discomfort such as diarrhea, vomiting, abdominal pain and/or loss of appetite.

### Samples

Owners were supplied with sterile collection tubes, gloves and directions for appropriate sample collection and storage. Freshly voided fecal samples were collected with minimal contamination and refrigerated prior to transportation to the laboratory (within 24 h), where a 250 mg aliquot was collected in a sterile manner and stored at −80°C in preparation for gDNA extraction. Samples were taken at baseline (visit 1), every 2–3 days during the treatment period (visits 2–5), then every 3 days (visits 6–8) after cessation of probiotic. A final sample was collected 3 weeks after completing treatment (visit 9).

### DNA Extraction

Fecal DNA was extracted using Power soil DNA isolation kit (MoBio laboratories, Catalog No. 12888-100). The 250 mg fecal aliquots were processed using the protocol for DNA isolation, as detailed in the manufacturer's instructions, with some modifications. Briefly, the fecal pellet was added to a glass bead tube (0.1 mm) and 750 μL of bead solution and 60 μL of C1 solution were added. Then, samples were incubated at 94°C for 10 min. Tubes were then placed in the PowerLyzer® 24 and were run at 3,000 rpm for 45 s. Extracted DNA was eluted from the spin column in 100 μL of C6 solution from Mobio (10 mM tris-Cl pH 8.0–8.5). Extracted DNA was quantified and checked for purity, based on UV absorption ratios 260:280 nm and 260:230 nm, on a ND1000 spectrometer. DNA extraction was repeated if the sample's 280/260 ratio was <1.65 or if DNA quantity was <10 ng/μL.

### Ion-Torrent Pyrosequencing

The extracted gDNA underwent ion torrent pyrosequencing, as described previously (26), using the universal PCR V4/5 region primers 515F and 806R at University of Western Australia School of Biomedical Sciences. Samples were sequenced in two runs (each run: 63 samples), the sequence depth was ~27,000 reads per sample.

Raw data was analyzed using the open-source software package Quantitative Insights into Microbial Ecology (QIIME), version 2 (QIIME2, release 2020.8) (27). Demultiplexing was carried out using the qiime2 plugin: qiime cutadapt demux-single (28) and quality filtering using the pipeline DADA2 (29). The plugin qiime dada2 denoise-pyro, denoises and clusters reads based on amplicon sequences variants (ASV, threshold 100% similarity). Taxonomy assignment to the unique sequences was done by using a pre-trained naïve Bayes classifier trained against Greengenes (13_8 revision) trimmed to contain only the V4 hypervariable region and pre-clustered at 99% sequence identity. A phylogenetic tree was generated using sep from the q2-fragment-insertion plugin (30). Samples with <500 reads (counts) and features (taxa) with a total abundance (summed across all samples) of <10 were removed. For posterior analysis, runs were merged.

### Statistical Analysis

Measurements of alpha (α)-diversity and beta (β)-diversity were performed using Microbiome and Phyloseq packages from R (31). The Shannon index was used to express α-diversity. Using the Bray-Curtis distance β-diversity was assessed (32). To calculate richness and α/β diversity indices, samples were rarefied at 7,367 sequences per sample for even depth of analysis. Rarefaction curves indicated that the rarefaction depth was appropriate for the analysis.

Hierarchical clustering was done using unweighted pair group method with arithmetic mean (UPGMA) method (hclust parameter method = “average”) and the Bray-Curtis distance.

Shannon index (α-diversity) was defined as the response in a linear mixed model, which included a subject-level random intercept. The model was defined using the “lme4” package in R (33). Informativeness of the model was assessed using the marginal and conditional coefficients of determination as implemented in the “muMin” package (34, 35). The package “emmeans” from R, was used for *post-hoc* comparisons among time points and for estimating marginal means and their 95% confidence intervals (36).

Associations between the sampling stage (baseline, probiotic, or post-administration), and the relative abundance of phyla or families, were assessed using a hierarchical multinomial regression model with the logit link function (37, 38), implemented in the “brms” package (39) in R. Observations from all time points were included, but only the stage of sampling was included in the model. Interpretation of effects was conducted from the posterior predictions of the population taxa abundances using 10,000 simulated reads, for uniformity.

## Results

### Animals

A total 15 adult dogs were enrolled in the study (Table 1). Subjects ranged from 3 to 11 years of age (mean 6.6 years; median 7 years), representing pure and mixed breeds weighing from 5 to 40 kilograms. There were seven castrated males, seven spayed females and one entire female.

**Table 1:** Signalment information of study population.

**Dog number**	**Breed**	**Age (years)**	**Sex/neutering status**	**Weight (Kg)**
1	Cavalier king charles spaniel X poodle	7	MN	5.5
2	Cavalier king charles spaniel X poodle	3	FS	6
3	Greyhound	5	FS	27
4	Boxer cross	10	MN	20
5	Kelpie cross	10	MN	20
6	Bull Harab X Catahoula Leopard dog	3	MN	40
8	Greyhound	5	FS	34
9	Rhodesian ridgeback cross	9	MN	35
10	Poodle cross	10	FS	10
11	Labrador retriever x dalmatian	8	FS	40
12	Terrier cross	11	MN	7.7
13	Golden retriever	4	FS	25
14	Beagle X cavalier king charles spaniel	9	FS	7.5
15	Cavalier king charles spaniel	3	MN	6.5
16	Gordon setter	3.5	FE	30

*M, male; F, female; S, spayed; N, neutered; E, entire*.

One participant missed a single sample collection during the trial (dog 8, visit 8), while dog 9 missed several sample collection dates and only submitted 3 samples in total [baseline (visit 1), during trial (visit 2) and after cessation of treatment (visit 9)]. Dog 11 submitted only the first four time-point samples and was removed from the study due to development of acute diarrhea following a known dietary indiscretion event. No other adverse gastrointestinal effects were recorded, and no changes in fecal consistency or frequency were observed over the entire study period.

### Effect of Probiotic on the Relative Abundance of Bacterial Phyla

From 123 samples, a total of 3,354,864 high-quality sequences were obtained, with the number of reads ranging from 236 to 55,535 per sample (median 26,537.5; mean 27,055.35; standard deviation (SD) 9,084.28).

**Figure 1:**
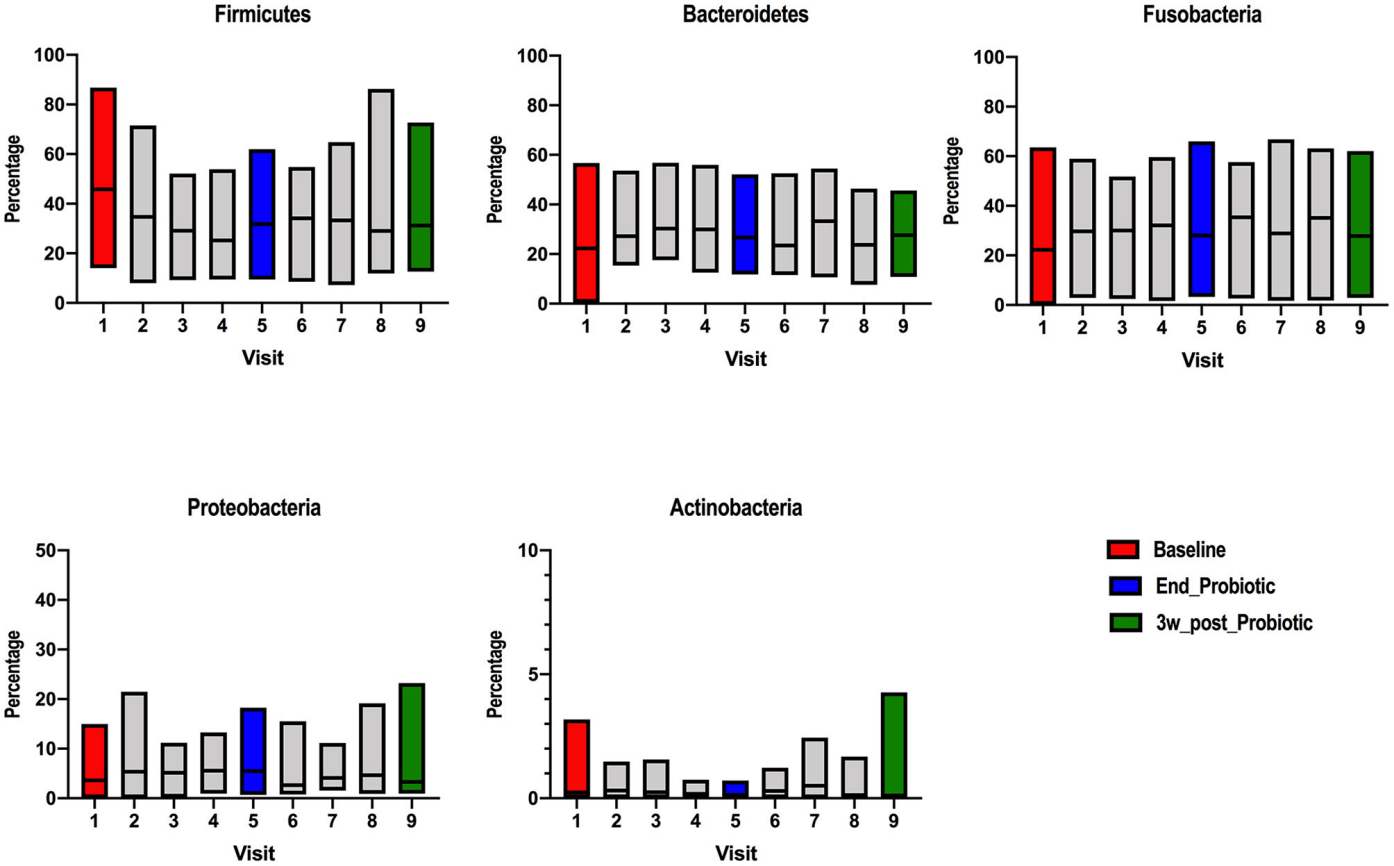
Relative abundance of different bacterial phyla detected on ion-torrent pyrosequencing at different stages of probiotic administration. Visit 1(red): baseline, visit 5 (blue): end of 2-week probiotic trial and visit 9 (green): samples taken 3 weeks after cessation of the probiotic.

The relative abundance of the different bacteria at phylum and family phylogenetic levels were compared among the different stages. In the raw data, the relative abundance was highly varied among stages (Supplementary Figure 1). At phylum level, Firmicutes, Bacteroidetes and Fusobacteria were the most populous (Figure 1), with minor proportions of Proteobacteria and Actinobacteria, as previously reported (40). At baseline, Firmicutes had a median abundance of 46% (range: 14–87%), Bacteroidetes 22% (range: 0.4–57%), Fusobacteria 22% (range: 0–63%), Proteobacteria 4% (range: 0–15%) and Actinobacteria 0.24% (range: 0–3%). At the end of the probiotic trial (visit 5), Firmicutes still predominated with a median abundance of 32% (range: 9–62%), followed by Fusobacteria 29% (range: 3–66%), Bacteroidetes 27% (range: 12–52%), Proteobacteria 6% (range: 0.7–18%), and Actinobacteria 0.13% (range: 0–0.71%). Finally, 3 weeks after cessation of the probiotic, Firmicutes relative abundance had a median of 31% (range: 13–73%), Fusobacteria 28% (range: 3–63%), Bacteroidetes 28% (range: 11–46%), Proteobacteria 3% (range: 1–23%), and Actinobacteria 0.11% (range: 0–4.27%). The same level of variation was seen at other phylogenetics levels. The relationship between the two dominant phyla, expressed as the Firmicutes to Bacteroidetes (F/B) ratio had the tendency to decrease during probiotic administration and after cessation (Supplementary Figure 2).

### Effects of Probiotic on Gut Microbial Alpha and Beta Diversity

**Figure 2:**
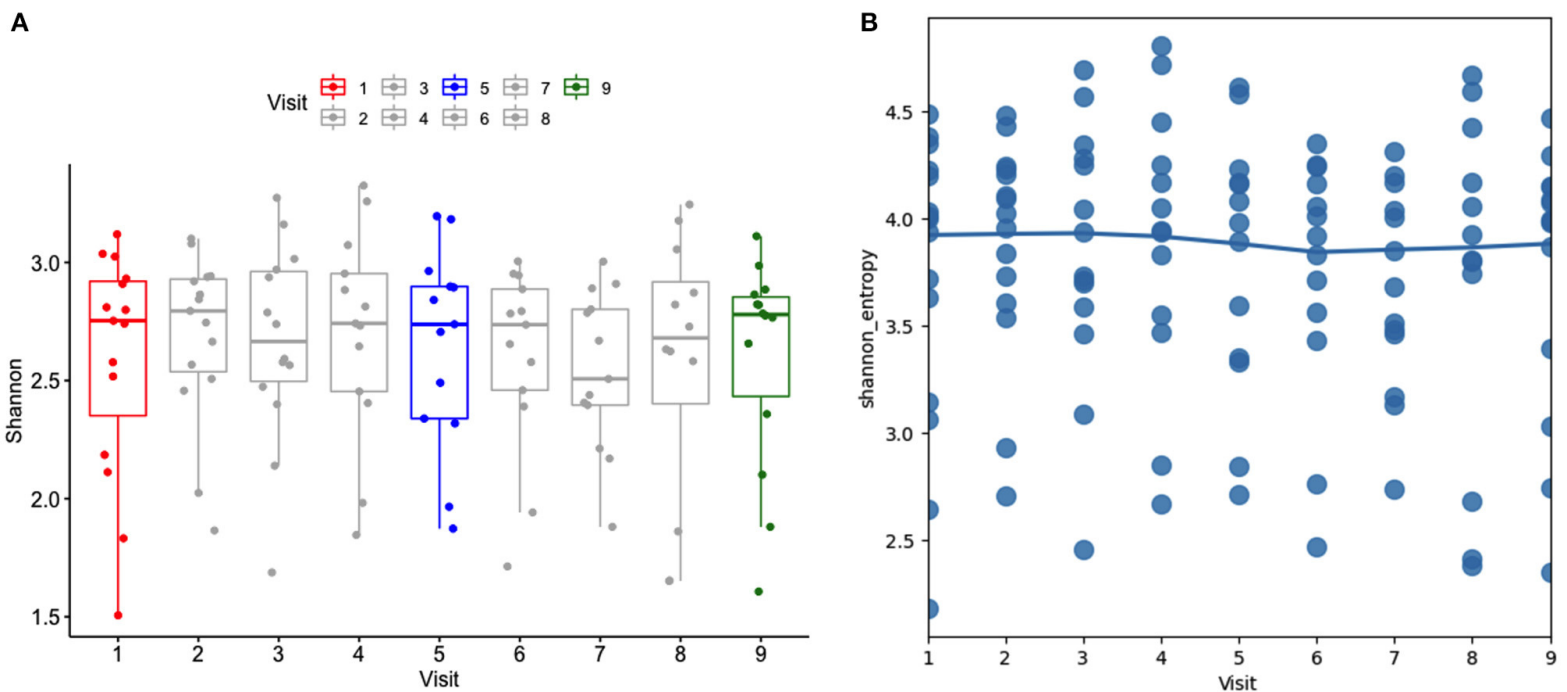
**(A)** Alpha diversity using the Shannon index at different stages (visits). Visit 1(red): baseline, visit 5 (blue): end of 2-week probiotic trial, and visit 9 (green): samples taken 3 weeks after cessation of the probiotic. **(B)** Regression scatter plot linear mixed effects model of alpha diversity demonstrating the trend of data.

Alpha diversity was analyzed using the Shannon index considering the subject as well as the stage. In general, Shannon diversity index was not affected by the administration of the probiotic when time and subject were considered (Figure 2A); although it was lower after cessation of the probiotic (Marginal R^2^: 0.013). The regression scatter plot (Figure 2B) demonstrates that the data is dispersed and there is no trend in diversity over time, such low variability may indicate an absence of consistent effect.

**Figure 3:**
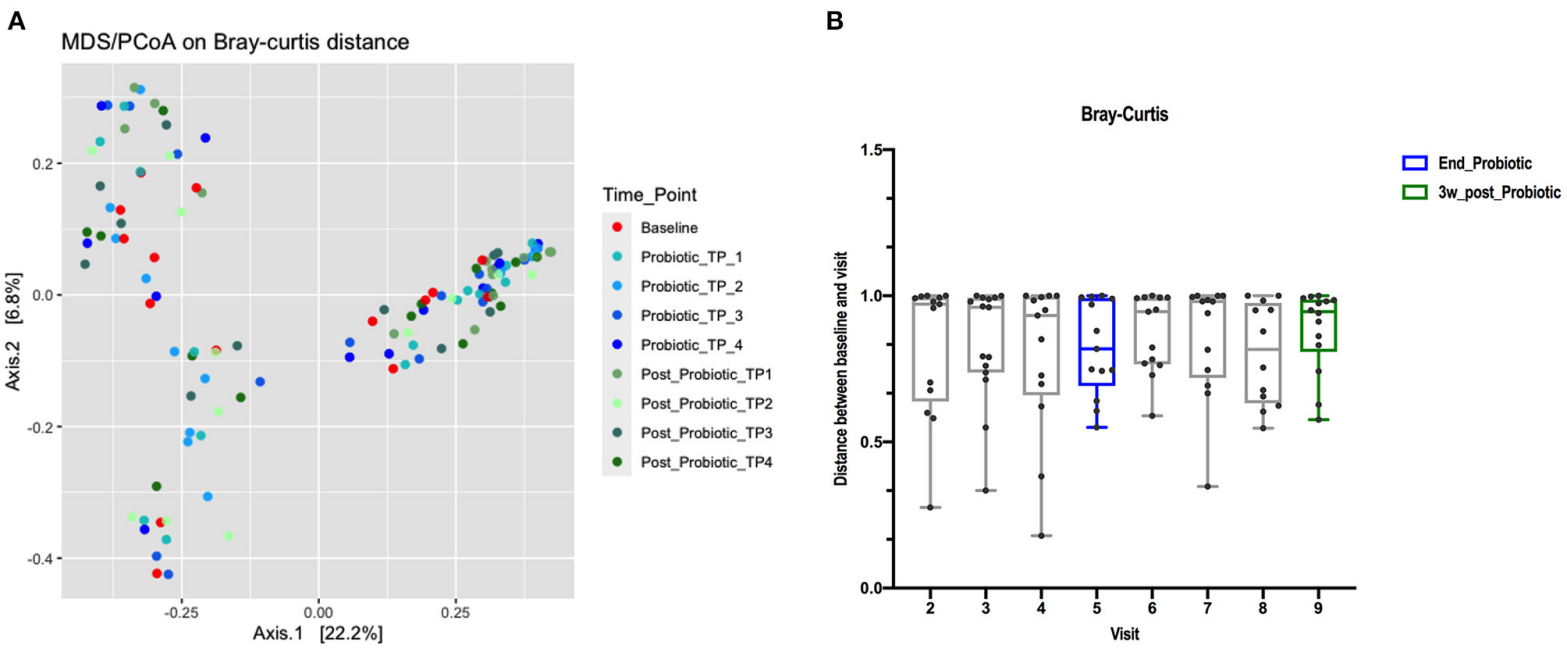
**(A)** PCoA using Bray-Curtis dissimilarity index. The percentage of variation explained by the principal coordinates (PC1 and PC2) is indicated on the axes (color of data points reflects stage of trial, red baseline, blues for during probiotic administration and greens for post-administration). **(B)** Distance boxplots of the differences in relative abundance between the baseline and the post-treatment sample from the same dog, measured as Bray-Curtis dissimilarity index.

Beta diversity principal coordinates analysis (PCoA) plots were constructed using a Bray-Curtis distance matrix at different stages of probiotic administration (Figure 3A). There was not a clear separation between baseline, the end of the probiotic administration and samples 3-weeks after cessation of the probiotic. Permutational multivariate analysis of variance (PERMANOVA) (Adonis) using the Bray-Curtis distance showed that the administration of probiotic explained 5% of the variability in β-diversity (R^2^: 0.04, *p*: 0.972). A detailed analysis per visit also did not show any clear separation (R^2^: 0.05, *p*: 0.955). Analysis of each group separately, showed that the shifts of the microbiota exhibited a great variability over time when compared to baseline, although, the distance decreased after the administration of the probiotic (indicating less variation) (Figure 3B).

### Hierarchical Cluster Analysis

Hierarchal clustering of samples based on their Bray-Curtis dissimilarity was performed using UPGMA clustering. We observed that samples have the tendency to cluster or be in proximity based on the subject (intra-individual) but not according to stage (time associated variability) (Supplementary Figure 3).

### Effect of Probiotic on Gut Microbial Composition at the Family Level

**Figure 4:**
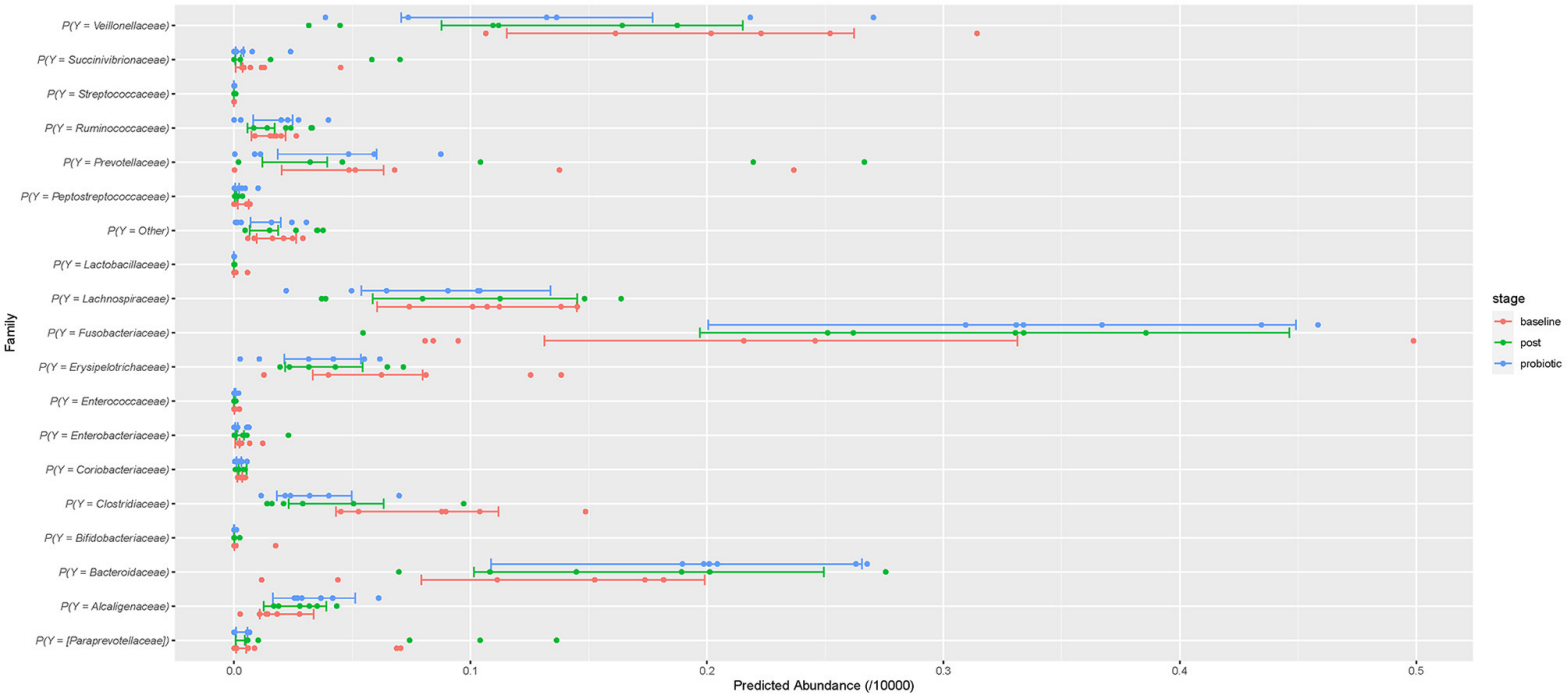
The predicted relative abundances at family level assuming 10,000 multinomial trials. The points are the individual-level predictions. The bars are the 50% credible intervals for the population prediction. Red represents baseline abundance, blue during probiotic administration, and green after cessation of the probiotic.

To assess how probiotic administration affected the canine GI microbial composition, a multinomial regression model was generated to compare the microbial differential abundance in each sampling stage while accounting for variation between dogs (Supplementary Table 1). The family level was chosen as the response because several microorganisms could not be classified at lower phylogenetic levels. A noticeable variation was observed among subjects and within each stage. Both during and after administration of the probiotic, *Clostridiaceae* was of lower abundance while *Fusobacteriaceae* and *Bacteroidaceae* were higher compared to baseline. These effects are weak, and the precision of estimation is low. They can be visually assessed from the predictions of the model (Figure 4) which summarizes effects present in the whole data, but direct interpretation from this model is difficult (37). Notably, the taxa of probiotic bacteria present in the supplement were of low abundance throughout all time points.

## Discussion

This study evaluated the effect of dietary supplementation with a commercial veterinary synbiotic on the intestinal microbiota of healthy dogs. The product from the present study, Paw DigestiCare 60™, contains six subspecies of lactic acid bacteria (LAB) (*Lactobacillus acidophilus; L. delbrueckii* subsp. *bulgaricus; L. lantarum; L. rhamnosus; Enterococcus faecium; Streptococcus alivarius* subsp. *Thermophiles*); and one strain of bifidobacteria (*Bifidobacterium bifidum)*, in addition to prebiotics. No adverse effects to the synbiotic were reported during the trial.

During administration of the synbiotic, the relative abundance of members of probiotic bacterial families varied over time and was highly individualized, suggesting variable degrees of probiotic survival and/or gut colonization. In general, synbiotic administration had a positive effect on abundance of *Enterococacceae* and *Streptococacceae* families, suggesting successful (at least temporarily) colonization or survival of GI transit. Meanwhile, *Bifidobacteriaceae* and *Lactobacillaceae* families remained unchanged or decreased during synbiotic administration. Relative abundances of *Enterococcus* and *Streptococcus* spp. returned to baseline after treatment discontinuation and no meaningful changes in the major bacterial phyla were identified with ion torrent-pyrosequencing.

In the present study, *Bifidobacteriaceae* members were detected in samples from only two dogs, and only one dog's sample detected members of the *Lactobacillaceae* family. *Enterococacceae* members were detected in three dogs and *Streptococacceae* in four dogs. The majority of known human and animal probiotic strains belong to *Lactobacillus* and *Bifidobacterium* genera (41). Lactobacilli commonly inhabit all parts of the GIT (42) ranging from 10^4^ to 10^8^ CFU/mL and among them, *L acidophilu*s is predominant (43).

Previous studies report that dog feces contain 10^8^ cells/g of *Bifidobacteria*, determined by fluorescence *in situ* hybridization (FISH) (44). The low abundance of *Bifidobacteria* genus in 16S rRNA gene surveys of diversity may be the result of it being a measure of relative abundance, not absolute. Gram-positive organisms (such as *Bifidobacteria*) can also be underrepresented in microbial profiling studies due to primer selection and their thick cell wall (45). Although the method of DNA isolation performed in this study (bead beating) is highly effective for the lysis of bacteria (46) and the primers used are reported to match >95% of the sequences in Ribosomal database project (RDP) from all the major bacterial phyla in the gut (Firmicutes, Bacteroidetes, Actinobacteria, Verrucomicrobia, and Proteobacteria) (47); additional research using quantitative polymerase chain reaction (qPCR) could be performed in addition to ion torrent pyrosequencing to corroborate these results.

The present study did not observe major changes at higher phylogenetic levels (Figures 1, 2), although the relative abundance of the Firmicutes phylum and the F/B ratio (Supplementary Figure 2) tended to decrease during and after administration. This observation is consistent with previous research showing that ingestion of probiotics does not alter the major bacterial phyla in feces (40, 48). At family level, the abundances of some members were altered in some individual dogs (Figure 4). Small changes in abundance were observed during and after administration of the synbiotic, *Fusobacteriaceae* and *Bacteroidaceae* families increased whereas the *Clostridiaceae* family exhibited a decrease in abundance. Due to trial design lacking the collection of more baseline samples, or a control population, we cannot conclude that the increase of bacterial families in feces were due to administration of the synbiotic alone and not simply normal variation. However, previous human and canine studies have also found that the use of a synbiotic caused an increase in the relative proportions of sequencing tags belonging to the phylum Fusobacteria at all phylogenetic levels down to the genus *Fusobacterium* when compared to baseline values (16, 40). Also, like ours, another study evaluating the quality of *L. acidophilus* DSM13241 as a feed additive in healthy adult dogs at a dose of 10^9^ CFU/mL for 4 weeks, found a decrease in absolute abundance of clostridial organisms in feces (using culture and fluorescence *in situ* hybridization) (49).

The clinical significance of these findings is unknown and warrants further investigation with longitudinal studies, in both healthy and diseased animals. They may indicate a potential therapeutic role for probiotics, as both the *Fusobacteriaceae* and *Bacteroidaceae* families have been noted to be of reduced abundance in animals with chronic enteropathy or inflammatory bowel disease when compared to healthy dogs (50–52).

The abundance of *Fusobacterium spp*. is used in the calculation of the dysbiosis index (along with *Faecalibacterium* spp., *Turicibacter* spp., *Streoptococcus* spp., *E. coli, Blautia* spp., *and Clostridium hiranonis*), with reduced abundance (and higher dysbiosis index score) reported in dogs with chronic enteropathies (53, 54). The dysbiosis index (DI) is a qPCR-based tool developed to assess fecal dysbiosis in dogs with chronic enteropathy, it utilizes bacterial taxa shown to be altered in studies of dogs with and without intestinal inflammation (53). The DI has only been evaluated in a small number of animals, with reported sensitivity of 74% and specificity of 95% using a threshold of DI of 0 as consistent with dysbiosis (53). It has not yet been demonstrated whether the DI is improved in response to management of the enteropathy.

In addition, the F/B ratio has been reported to play an important role in maintaining intestinal homeostasis, with increases or decreases often associated with obesity and with IBD, respectively (55, 56). Although, other reports have shown an opposite trend. Due to diverse lifestyle-associated factors, the relative abundance of the Firmicutes and Bacteroidetes phyla is highly variable between subjects from a same population, and we should bear in mind that this ratio can be affected by an increase in other phyla and that dysbiotic increases in other phyla do not necessarily change the F/B ratio (55). Future investigation of probiotics may benefit from comparing microbial diversity using the DI and comparing the F/B ratios in addition to other diversity analysis, particularly where probiotic administration is being used to treat enteropathy or obesity.

The observations found in the present study agree with a similar study which used a different multi-strain synbiotic formulation (containing *Bifidobacterium, Enterococcus, Streptococcus*, and four strains of *Lactobacillus* spp. totaling 5 × 10^9^ CFU) administered to healthy dogs for 21 days. They reported no alteration of the fecal microbiota abundances at higher phylogenetic levels during or after administration, but the proportion of *Fusobacteria* were found to be significantly increased during synbiotic administration (40). Also, although this study reported detection of all probiotic species in the feces of dogs; only *Enterococcus* and *Streptococcus* counts increased significantly in at least one time point during administration and returned to baseline abundance after treatment was discontinued. Like our own study, the detection of probiotic species was not equal among different individuals. Interestingly, at baseline the most abundant phylum was Firmicutes but the second must abundant was Actinobacteria which was least abundant in the present study (with Bacteroidetes being second most abundant). This difference may be a result of geographic differences of microbial diversity, differences in sample treatment and genome sequencing or a combination of factors (57). Such differences in baseline microbial diversity may impact whether a host microbiome is resistant or permissive to probiotic colonization and effects (58).

Often, effects of a probiotic are seen only for the duration of administration with changes to microbiological composition reverting to initial diversity within days of cessation of treatment. Here, we observed that the levels of the families of the probiotic strains returned to baseline levels only, after 3 days of cessation of the probiotic (Figure 4). In humans, strain- and person- persistence variability ranges from 2 days up to 6 months following cessation of probiotic supplementation (10, 59).

In dogs, some studies have found that the effects of a probiotic occur only for the duration of administration, with changes to microbial composition reverting to initial diversity within days of cessation of treatment (9, 40, 49). Whereas, other studies, have found persistence of the probiotic strain after discontinuation of the product. In one study, *L. rhamnosus* GG (LGG), administered to dogs at 5 × 10^11^ CFU per day, was recovered from feces in quantities reflective of the dose administered and persisted for 4 days after discontinuation (60). Likewise, a study using a canine-originated probiotic strain *Enterococcus faecium* EE3, administered for 1 week to 11 healthy dogs at a dose of 10^9^ CFU/mL, found the strain persisted in feces for 3 months after cessation of its administration at a level of 6.83 ± 0.95 log CFU/g (61). This inconsistency likely reflects that probiotic strain, dose, duration of treatment and the host's resident microbiome may all affect probiotic persistence (10). Also, direct comparison between relative (qPCR) and absolute abundance (culture) cannot be made, but these discrepancies indicate a need for future studies to consider the measurement of both relative and absolute abundance of probiotic strains (12, 62).

The phylogenetic coverage of 16S rRNA gene clone libraries is generally limited to the species level (42) impeding the distinction of probiotic strains and endogenous bacteria that are closely related (10); and cannot confirm the viability of the bacteria (bacterial DNA from viable and nonviable microorganisms is detected) (63). Further, the presence of the bacterial DNA in feces may only indicate passage of the microbes through the GIT and their subsequent excretion but does not indicate true GI mucosal colonization. Thus, in future studies, complementing sequencing with culture, mucosal biopsy, qPCR and/or analysis at strain level (shot gun metagenomics) would be ideal, as well as comparison of changes at the mucosal surface with feces.

Another consideration when investigating microbial diversity is that due to the low abundance of the targeted probiotic groups, changes may not be evident at taxonomic level but may exist at a functional level (12). Bacterial metabolites and/or bacterial components (including DNA) from the synbiotic may be present at the mucosal surface and can influence biological function, regardless of changes in taxonomy (12, 13). More studies assessing genetic capability of the bacteria (shotgun metagenomics, transcriptomics), impact of bacteria on clinicopathological parameters, immune system function, metabolic functions (metabolomics) and amino acid production (proteomics) would help to elucidate the biological changes in the GI microbiome. The investigation of probiotic/prebiotic impact on bacterial and host metabolomics in health and disease, will likely be more important in future research than changes to microbiome taxonomy alone. Finally, an increase in the 16S rRNA gene relative abundance of the administered probiotic strain should not be interpreted unequivocally as an effect on the GI microbiome. As supplemented probiotic bacteria are excreted in the feces, increase in their relative abundance concomitantly will lead to a reduction in the relative, but not absolute, abundance of other community members. As such, over interpretation of the significance of change in abundance should be avoided (7, 62).

Previously, it was thought that the bacterial species used in probiotics should be of host species intestinal origin to produce beneficial health effects (9, 64). This was in part because it was assumed that the ability to adhere to the intestinal mucosa, to some extent, was host-species specific. However, probiotic strains of human origin intended for human use have been shown to adhere to canine intestinal mucosa, indicating that species-specificity may not always be necessary for adhesion (65). Both dog-associated (e.g., *Bifidobacterium pseudolongum* and *Bifidobacterium animalis*) and human associated (e.g., *Bifidobacterium catenulatum* and *Bifidobacterium bifidum*) strains of bifidobacteria have been found in dog feces (44, 66, 67).

Limitations of this study include the small number of dogs with variable ages and breeds, short duration of treatment, and different home environments, which may have prevented detection of differences between treatment stages. As an open-label study with no placebo group it is possible that any observed changes in microbiota were a result of normal individual variation (6). The baseline sample collected from subjects in this study was performed so that each dog would serve as its own control removing the need for a control group comparison (68). Based on the high individual variation noted in our study, a longer period of baseline sample collection may have provided a better baseline for comparison and should be considered for future investigations. As baseline microbiota composition varies between dogs, longitudinal studies allow a better assessment not only of community stability but also shifts in specific taxa. Use of a separate group of control dogs may also provide useful information on the natural variation of fecal microbial diversity of individual dogs but given the large number of uncontrolled variables between client-owned dogs such controls may not be directly comparable to subjects in a treatment trial.

Moreover, as the diet of subjects was not controlled (although kept constant for at least 2 months prior to and during the trial), variable macro and micronutrient intake could have influenced the effect of the probiotics on host microbiome diversity. However, longitudinal studies (as used here) have been shown to be optimal in determining the impact of an intervention on the GI microbiome in humans, due to highly individualized baseline microbiota and effect of dietary variation (62, 68). Furthermore, recent studies in dogs have shown that the kingdom of origin of the ingredients seems to be less important than the overall macronutrient composition of diet. Extruded diets with similar macronutrient contents, prepared exclusively with vegetable sources of protein, did not significantly alter the microbiome of healthy dogs (69–71).

Recently, a prospective, placebo-controlled study in a population of German Shepherds and Belgium Shepherds at a training base, demonstrated that the strongest response to administration of a probiotic preparation was observed in the group of elderly dogs (>5 years), compared with training and young dogs (72). The gut microbiota of older dogs was shifted to a young-like composition during administration of the probiotic formulation (a three-strain compound of 2 × 10^9^ CFU, not commercially available), but reverted to an “elderly microbiota age index” within 15 days of stopping the treatment (72). This study also implemented use of general health markers, feed intake and weight gain, which improved in the treatment group compared to the control. In our study, the dogs were a range of ages and breeds which may have contributed to the greater variation noted between individuals, however no discernible difference in response was seen between older and younger dogs (which may be a result of small sample sizes). Future studies of healthy dogs should aim to assess the response of the microbiome in different age and breed groups, as well as use measurable outcomes relevant to the host, such as improved appetite or weight gain, rather than reporting microbiome features alone.

The quantity of each probiotic present in the product studied is not specified on the label, which will complicate future comparisons with other formulations. A duration of administration of 2 weeks was chosen to maximize owner compliance with the trial, and previous studies have reported changes in fecal microbial composition within similar time periods (40, 73, 74). An optimal period of administration has not been determined for healthy canine or human patients, but it is possible that a longer duration may result in more durable or significant changes. Likewise appropriate dosage of probiotics remains unclear, most trials utilize different formulations containing various concentrations of a variety of probiotic organisms which makes direct comparison challenging especially due to the absence of standardized markers for efficacy. In this study the formulation is available commercially (whereas other studies utilize custom made products with known bacterial counts), which meant that the dosage of each probiotic strain was unknown, and the total dose of 6 × 10^7^ CFU per two grams of product is less than total doses reported in previous probiotic trials. It is also notable that previously the label accuracy of veterinary probiotic products in Canada was found to be highly variable (75).

Furthermore, it would be useful to assess the viability and concentration of the probiotic strains within the product at different time points during the trial. An evaluation of label accuracy and bacterial content in 25 commercially available products revealed that the overall level of bacterial growth was highly variable, with one product having no viable growth despite its labeling of 14 million CFU/capsule, and another product containing greater than the stated concentration of *Bifidobacterium animalis* (75). Likewise, a study investigating the effect of a synbiotic in healthy sled dogs showed that the microbial analysis detected viable organisms in the synbiotic at baseline, before implementing treatment; but at the end of the trial, only *Enterococcus* spp. could be cultured from the synbiotic supplement (74).

Paw DigestiCare 60™ also contains prebiotics and fermentable fibers such as spirulina, quinoa and alfalfa in addition to probiotics, which can impact the GI microbiome on their own (25, 76). Changes observed after administration could be due to the probiotics (including their concentrations relative to one another), the prebiotics, or an effect from the combination of these. Future studies evaluating the effects of these factors separately, and their interactions, are indicated.

## Conclusion

The response of an individual's microbiome to the administration of probiotics is dependent on factors related to the host, their environment, their existing microbiome, the product composition, duration of administration and dose. As such, subjects respond very differently to the same interventions. In this study, the administration of Paw DigestiCare 60™ lead to transient changes in the prevalence of some bacterial families for some individual dogs; these alterations were not persistent after cessation of treatment and there was no significant change in overall diversity at any stage in the trial. The longitudinal design of the study enabled observation that changes were highly variable both within and between individuals and between stages of the trial, a reflection of the natural inter and intra-individual microbiome variability with time. Future longitudinal studies investigating the functional changes to the microbiome (i.e., metabolomics, DI, and markers of general health), are indicated to understand the impact of synbiotic products on host intestinal function, both in health and disease.

## Data Availability Statement

The datasets presented in this study can be found in online repositories. The names of the repository/repositories and accession numbers can be found below: https://www.ncbi.nlm.nih.gov/, PRJNA680042.

## Ethics Statement

The animal study was reviewed and approved by University of Melbourne Animal Ethics Committee (ID1413272.2). Written informed consent was obtained from the owners for the participation of their animals in this study.

## Author Contributions

CM: conceived and designed the experiment. SC: performed the experiments. RA and LM-L: microbial data analysis. LM-L and AW: statistical analysis. SC, LM-L, AW, RA, and CM: drafting the paper and paper revisions and final approval. All authors contributed to the article and approved the submitted version.

## Conflict of Interest

The authors declare that the research was conducted in the absence of any commercial or financial relationships that could be construed as a potential conflict of interest.
