# Supplementary material for: Longitudinal Survey of Fecal Microbiota in Healthy Dogs Administered a Commercial Probiotic
Source: Front Vet Sci. 2021 Jun 21;8:664318. doi: 10.3389/fvets.2021.664318 (PMC8255976; doi:10.3389/fvets.2021.664318)
Supplement: Supplementary file 1 [file Table_1.DOCX]

| **Baseline** |  |  |  | **Probiotic** |  |  |  | **Post** |  |  |  |
| --- | --- | --- | --- | --- | --- | --- | --- | --- | --- | --- | --- |
| **Estimate** | **Q25** | **Q75** | **Family** | **Estimate.1** | **Q25.1** | **Q75.1** | **Family.1** | **Estimate.2** | **Q25.2** | **Q75.2** | **Family.2** |
| 0.000159 | 0.00012 | 0.000192 | P(Y = Bifidobacteriaceae) | 2.97E-05 | 2.16E-05 | 3.66E-05 | P(Y = Bifidobacteriaceae) | 0.000109 | 7.95E-05 | 0.000133 | P(Y = Bifidobacteriaceae) |
| 0.002906 | 0.001327 | 0.003541 | P(Y = Coriobacteriaceae) | 0.002523 | 0.001122 | 0.003085 | P(Y = Coriobacteriaceae) | 0.004351 | 0.001935 | 0.005298 | P(Y = Coriobacteriaceae) |
| 0.152525 | 0.079163 | 0.199091 | P(Y = Bacteroidaceae) | 0.200965 | 0.108601 | 0.265476 | P(Y = Bacteroidaceae) | 0.189285 | 0.101357 | 0.249513 | P(Y = Bacteroidaceae) |
| 0.051288 | 0.02013 | 0.063241 | P(Y = Prevotellaceae) | 0.048427 | 0.01844 | 0.060259 | P(Y = Prevotellaceae) | 0.032151 | 0.011927 | 0.039328 | P(Y = Prevotellaceae) |
| 0.006011 | 0.000794 | 0.00521 | P(Y = [Paraprevotellaceae]) | 0.006603 | 0.000867 | 0.005734 | P(Y = [Paraprevotellaceae]) | 0.005378 | 0.000696 | 0.004592 | P(Y = [Paraprevotellaceae]) |
| 0.000141 | 2.60E-05 | 0.000146 | P(Y = Enterococcaceae) | 0.000706 | 0.00013 | 0.000733 | P(Y = Enterococcaceae) | 2.41E-05 | 4.35E-06 | 2.48E-05 | P(Y = Enterococcaceae) |
| 5.39E-06 | 1.90E-08 | 2.17E-06 | P(Y = Lactobacillaceae) | 1.96E-06 | 6.92E-09 | 7.96E-07 | P(Y = Lactobacillaceae) | 3.72E-07 | 1.28E-09 | 1.51E-07 | P(Y = Lactobacillaceae) |
| 1.31E-05 | 5.23E-07 | 7.64E-06 | P(Y = Streptococcaceae) | 3.78E-05 | 1.53E-06 | 2.32E-05 | P(Y = Streptococcaceae) | 7.59E-06 | 3.03E-07 | 4.60E-06 | P(Y = Streptococcaceae) |
| 0.087861 | 0.04315 | 0.111813 | P(Y = Clostridiaceae) | 0.040055 | 0.018057 | 0.049808 | P(Y = Clostridiaceae) | 0.050515 | 0.023145 | 0.063263 | P(Y = Clostridiaceae) |
| 0.112204 | 0.06051 | 0.144913 | P(Y = Lachnospiraceae) | 0.103932 | 0.053815 | 0.133914 | P(Y = Lachnospiraceae) | 0.112498 | 0.058721 | 0.145175 | P(Y = Lachnospiraceae) |
| 0.005298 | 0.001626 | 0.00628 | P(Y = Peptostreptococcaceae) | 0.001817 | 0.000544 | 0.002105 | P(Y = Peptostreptococcaceae) | 0.000975 | 0.000292 | 0.001137 | P(Y = Peptostreptococcaceae) |
| 0.017796 | 0.007277 | 0.021703 | P(Y = Ruminococcaceae) | 0.019911 | 0.008028 | 0.024772 | P(Y = Ruminococcaceae) | 0.014 | 0.00561 | 0.017148 | P(Y = Ruminococcaceae) |
| 0.201741 | 0.115337 | 0.262261 | P(Y = Veillonellaceae) | 0.136436 | 0.070646 | 0.177006 | P(Y = Veillonellaceae) | 0.164164 | 0.08772 | 0.215192 | P(Y = Veillonellaceae) |
| 0.062365 | 0.033289 | 0.079747 | P(Y = Erysipelotrichaceae) | 0.041975 | 0.021275 | 0.053679 | P(Y = Erysipelotrichaceae) | 0.04281 | 0.021675 | 0.054435 | P(Y = Erysipelotrichaceae) |
| 0.245732 | 0.131215 | 0.331356 | P(Y = Fusobacteriaceae) | 0.333873 | 0.200512 | 0.449151 | P(Y = Fusobacteriaceae) | 0.330461 | 0.197078 | 0.446246 | P(Y = Fusobacteriaceae) |
| 0.027662 | 0.010945 | 0.033735 | P(Y = Alcaligenaceae) | 0.041695 | 0.016509 | 0.051235 | P(Y = Alcaligenaceae) | 0.031845 | 0.012476 | 0.039003 | P(Y = Alcaligenaceae) |
| 0.003346 | 0.000649 | 0.003597 | P(Y = Succinivibrionaceae) | 0.003737 | 0.00072 | 0.004032 | P(Y = Succinivibrionaceae) | 0.002673 | 0.000508 | 0.002848 | P(Y = Succinivibrionaceae) |
| 0.002 | 0.000579 | 0.002257 | P(Y = Enterobacteriaceae) | 0.001466 | 0.000418 | 0.001659 | P(Y = Enterobacteriaceae) | 0.003729 | 0.001076 | 0.004264 | P(Y = Enterobacteriaceae) |
| 0.020948 | 0.009614 | 0.026245 | P(Y = Other) | 0.01581 | 0.006945 | 0.019732 | P(Y = Other) | 0.015023 | 0.0066 | 0.018655 | P(Y = Other) |

**Supplementary Table 1:** Results of multinomial regression model comparing the microbial differential abundance between trial stages.
