# Supplementary figures and images for: Longitudinal Survey of Fecal Microbiota in Healthy Dogs Administered a Commercial Probiotic
Source: Front Vet Sci. 2021 Jun 21;8:664318. doi: 10.3389/fvets.2021.664318 (PMC8255976; doi:10.3389/fvets.2021.664318)

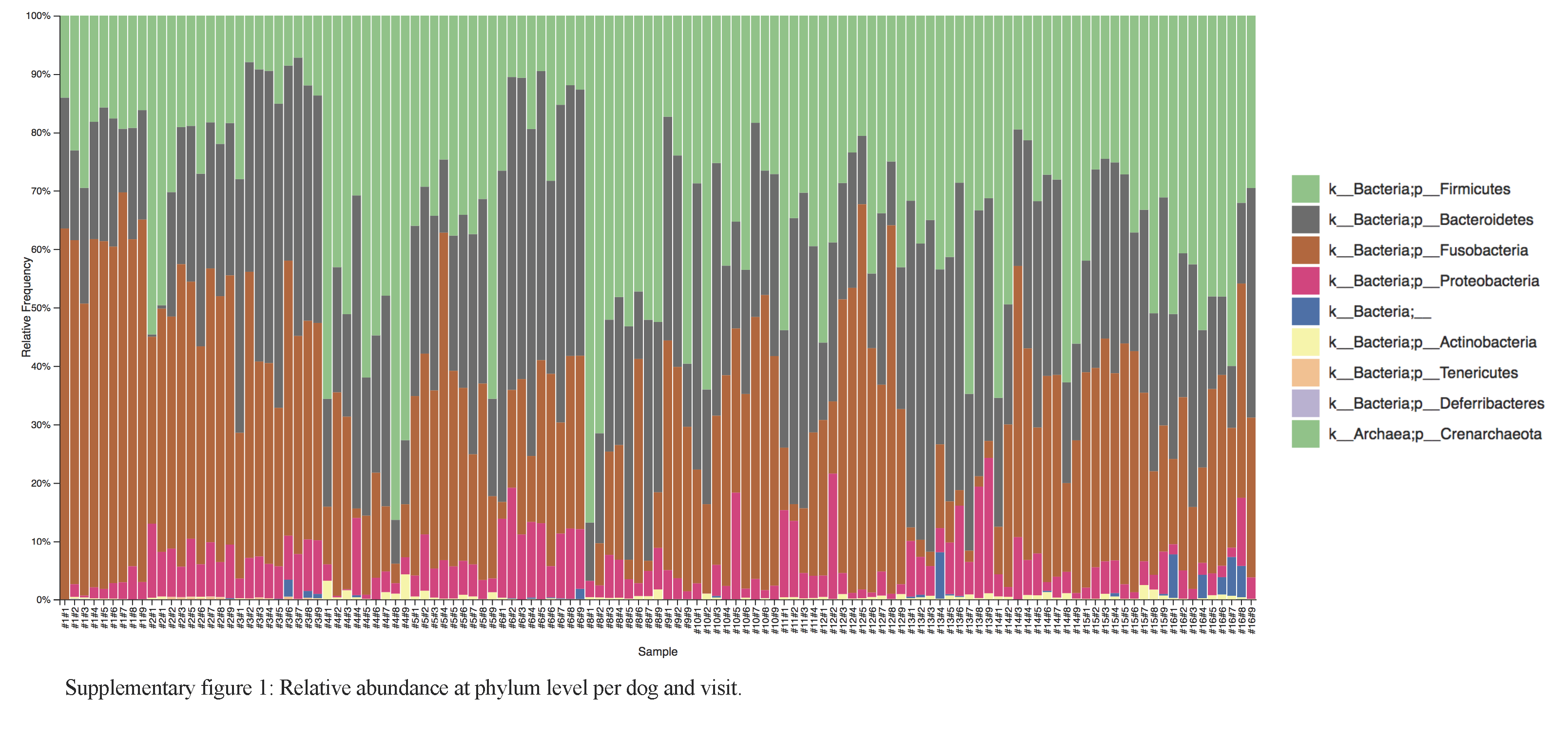

Supplement: Supplementary file 2 [file Image_1.TIFF]

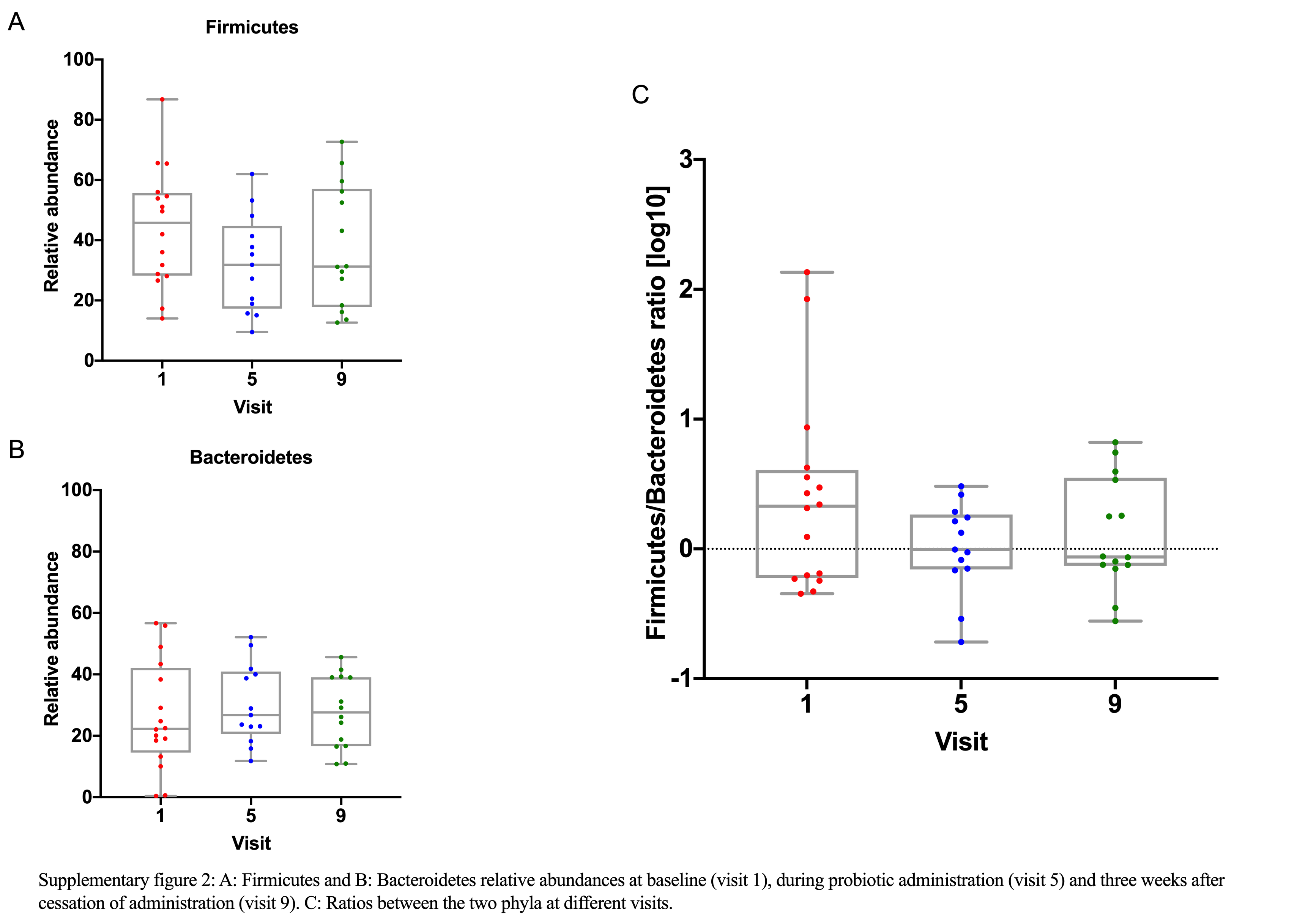

Supplement: Supplementary file 3 [file Image_2.TIFF]

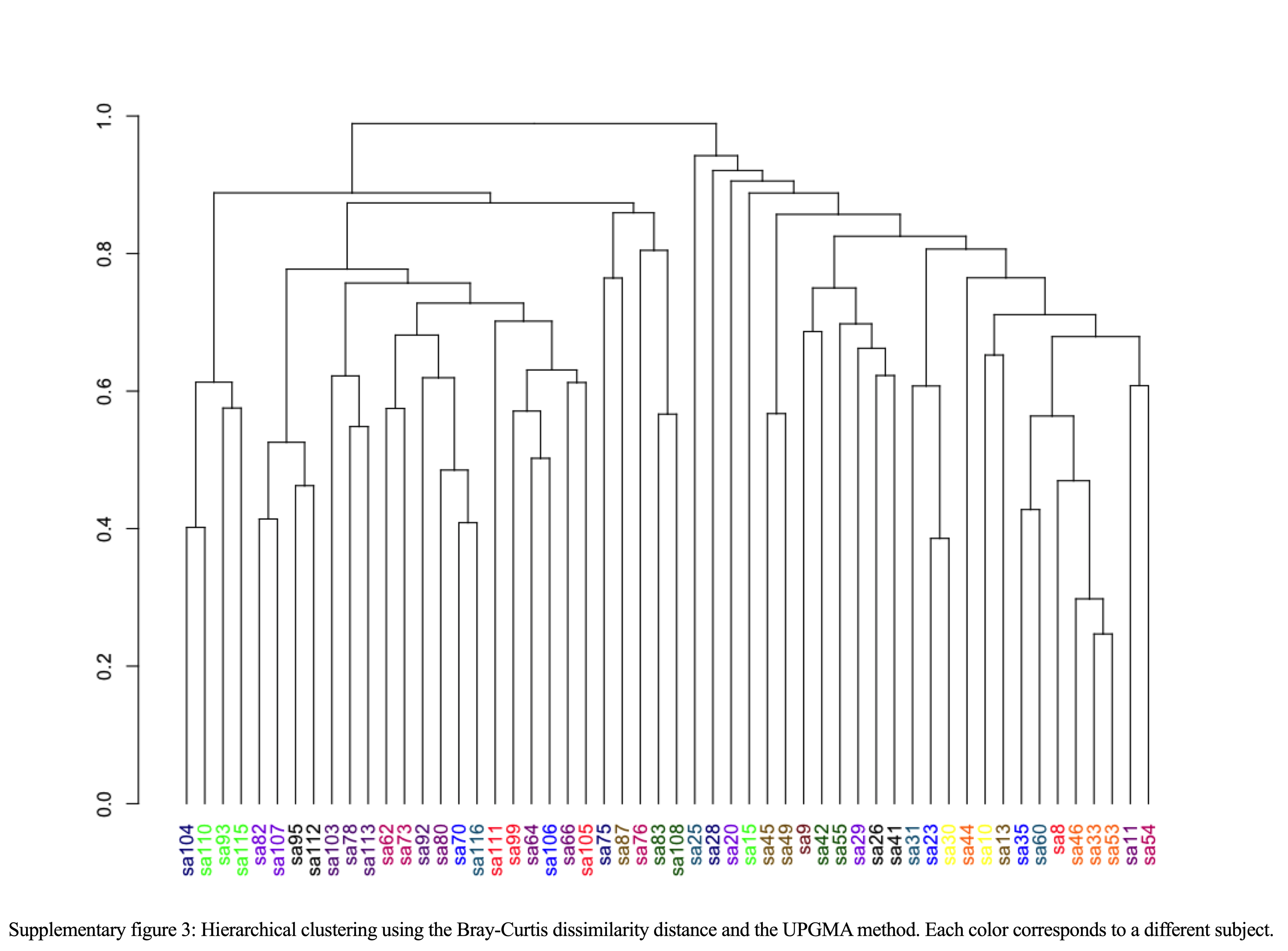

Supplement: Supplementary file 4 [file Image_3.TIFF]
